# Applying laser induced breakdown spectroscopy (LIBS) and elemental imaging on marine shells for archaeological and environmental research

**DOI:** 10.1038/s41598-023-46453-w

**Published:** 2023-11-13

**Authors:** Niklas Hausmann, Danai Theodoraki, Victor Piñon, Panagiotis Siozos, Andreas Lemonis, Demetrios Anglos

**Affiliations:** 1 Leibniz Zentrum für Archäologie (LEIZA), Ludwig-Lindenschmit-Forum 1, 55116 Mainz, Rhineland-Palatinate Germany; 2 Institute of Electronic Structure and Laser (IESL), Foundation for Research and Technology-Hellas, N. Plastira str. 100, 70013 Heraklion, Crete Greece; 3 Department of Chemistry, University of Crete, 70013 Heraklion, Crete Greece

**Keywords:** Environmental sciences, Chemistry, Optics and photonics, Physics

## Abstract

Using LIBS for the analysis of archaeological and geological marine mollusc shells is a growing research area that relies on customised instrumentation and specific workflows that can accommodate the variety and precision of the required sampling parameters. However, the increased efficiency offered by LIBS, which enables the study of a larger quantity of shell samples for temperature variation, ecological parameters, and human consumption practices, outweighs the initial efforts required to develop customised instrumentation and workflows. In this work, we present detailed specifications and parameters for the development of a LIBS system capable of generating Mg/Ca images on marine shells that directly correlate with seasonal sea temperatures. Our main objective was to develop specifications that enable easy adaptation of LIBS systems to existing laboratories for studying hard-tissue samples. These specifications were used to develop a customised micro-LIBS system and apply it to a real-world example of an archaeological study to better understand its efficiency on the marine mollusc shells and demonstrate its potential for broader applications in interdisciplinary research. In total 101 shell specimens have been analysed within a time frame of approximately 71 h of machine time, producing 234 images (100 µm resolution: 100 images, 30 µm resolution: 134 images). SEM analysis of the irradiated sections of the shell revealed a primary ablated area of 10–15 µm in diameter, while a secondary affected area of the shell’s crystal fabric extended to 30–50 µm after repeated shots. Overall, this new customised system reliably and efficiently analysed marine mollusc specimens without major destructive effects, enabling additional analyses for other proxies to be carried out. This study highlights the potential of the LIBS method for interdisciplinary research, encompassing applications in paleoclimatology, marine ecology, and archaeology.

## Introduction

The analysis of mollusc shell carbonate using geochemical methods has many uses in the geosciences and in archaeology for the study of past environments. Their preservation on geological time scales, with large volumes resulting from human predation for over 100,000 years and varied life-spans ranging from years to centuries, make them ideal archives for studying short-to-long-term environmental conditions on land and underwater. For archaeologists it is particularly important to gain information from a large number of shells, to best cover different parts of one archaeological site, which might represent different time periods, different areas of activity, different degrees of preservation. Specifically the use of information regarding the season of death is becoming increasingly important.

Extracting information from these archives requires a well-planned sampling strategy, as each mollusc species or even individual has a particular growth structure and needs a tailored approach when it comes to accurately describing its climate record. While some methods employ the extraction of shell carbonate via drills combined with the use of microscopic assessments of the growth structure^1–3^, other methods, mainly based on laser ablation, employ in-situ sampling of the carbonate, to better control sample origin or reduce the time of preparation or analysis^4,5^.

LIBS (Laser Induced Breakdown Spectroscopy) has started to establish itself as an alternative to traditional methods such as LA-ICP-MS (laser ablation—inductively coupled plasma—mass spectrometry) due to its fast data acquisition speed and minimal sample pre-treatment^6–9^.

Despite its demonstrated potential, LIBS has not yet become broadly accepted and applied as a fast screening method for analysing vast numbers of mollusc shells. There are two main reasons for the method's limited acceptance. Firstly, there is a lack of specific protocols that can be implemented by archaeologists and scientists in the fields of paleo-environment and archaeology. Secondly, there is a wide variety of analytical parameters associated with the LIBS apparatus and measurement conditions. Consequently, the absence of optimisation hinders the integration of the LIBS method into laboratory procedures.

Enigmatic physiological influences and compositional variations within isochronous parts of the shell can distort the environmental record. Understanding the patterns behind these influences will improve data interpretation and lead to the development of new climate proxies. Recent studies of our group^8,10,11^ have shown for the first time that extensive spatial imaging of multiple mollusc specimens using LIBS across a wider region can resolve the enigmatic patterns within the elemental record.

While traditional methods are based on the analysis of a line of individual or continuous sampling positions that follow the direction of growth, the fast data acquisition speed of LIBS also allows obtaining two-dimensional images. Imaging allows a more comprehensive analysis of the shell-record, as the location of the measurement does not need to be pre-determined through the additional evaluation of the growth structure using microscopes. As a result, any errors that occur due to inadequate sampling locations can be avoided. These errors occur often in the study of elemental ratios in mollusc shells^12–14^, where additional factors beyond external environmental conditions influence the elemental concentration with the potential to skew climatic reconstructions.

Based on these findings we have developed specific protocols for preparing samples to generate 2D elemental Mg/Ca images of whole limpet shells (e.g. *Patella* sp.). These protocols have been designed to be easily replicable by archaeologists and palaeontologists allowing for enhanced pre-screening of mollusc specimens, improved selection for stable isotope analysis, and ultimately, increasing the efficiency of isotope analysis by up to two orders of magnitude^10^.

Past studies of elemental analysis of mollusc shells using LIBS have employed a range of analytical parameters such as different spectral lines, resolutions, and spectra per location, but were in agreement that a fast acquisition speed is preferable and that an accurate control of the sample location is a main requirement^5,7,15^, the latter of which being less prioritised in very fast imaging systems e.g.^16^.

In this study, we built on previous work that aimed to rapidly map the elemental composition (i.e. Mg/Ca ratios) of marine mollusc shell sections^8^, with the goal of (a) increasing the speed of the analysis, (b) improving user-friendliness, and (c) producing more highly-resolved elemental images. Improving these features results in higher quality data and a wider range of potential applications and users. At the same time, we wanted to minimise the amount of material analysed and removed from the samples, to allow for further analyses in the future. The impact on the shell surface of several different sampling parameters was studied using SEM analysis.

By optimising the parameters of the LIBS apparatus to generate Mg/Ca images of shells, we provide guidelines that can be replicated for constructing setups for shell analyses. These setups can be seamlessly integrated into existing laboratories.

As marine mollusc shells are our main research interest, we are using a collection of samples of the species *Patella caerulea* (Linnaeus, 1758) acquired from 17 locations around Greece at different times during 2021 as part of an ongoing archaeological and climatological study of the region.

## LIBS system and methodology

### Mg and Ca emission line selection

In our past study^10^, we analysed various Ca and Mg emission lines in the 250 to 600 nm spectral range to identify the most reliable Mg/Ca ratio for marine shells. Two essential criteria had to be met by both Ca and Mg emission lines. First, the lines needed to fall within a narrow spectral range (~ 50 nm) recordable by a Czerny-Turner spectrometer with adequate resolution (< 0.2 nm). Although longer spectral distances could yield more accurate correlations between Mg/Ca and seasonal sea temperature, they require Echelle spectrometers with lower acquisition rates. Rapid spectra acquisition is crucial for the practical application of LIBS in marine shell studies. Second, the line intensity, particularly for Mg, must be as high as possible to ensure clear signals even at minimal Mg concentrations.

Our investigation revealed that the Ca II (2D3/2 → 2P1/2) emission line at 315.887 nm and the Mg II (2P3/2 → 2S1/2) emission line at 279.553 nm satisfy both criteria. We recommend using these two lines for accurate Mg/Ca ratio determination in marine shells with LIBS.

### Precursor system

**Figure 1:**
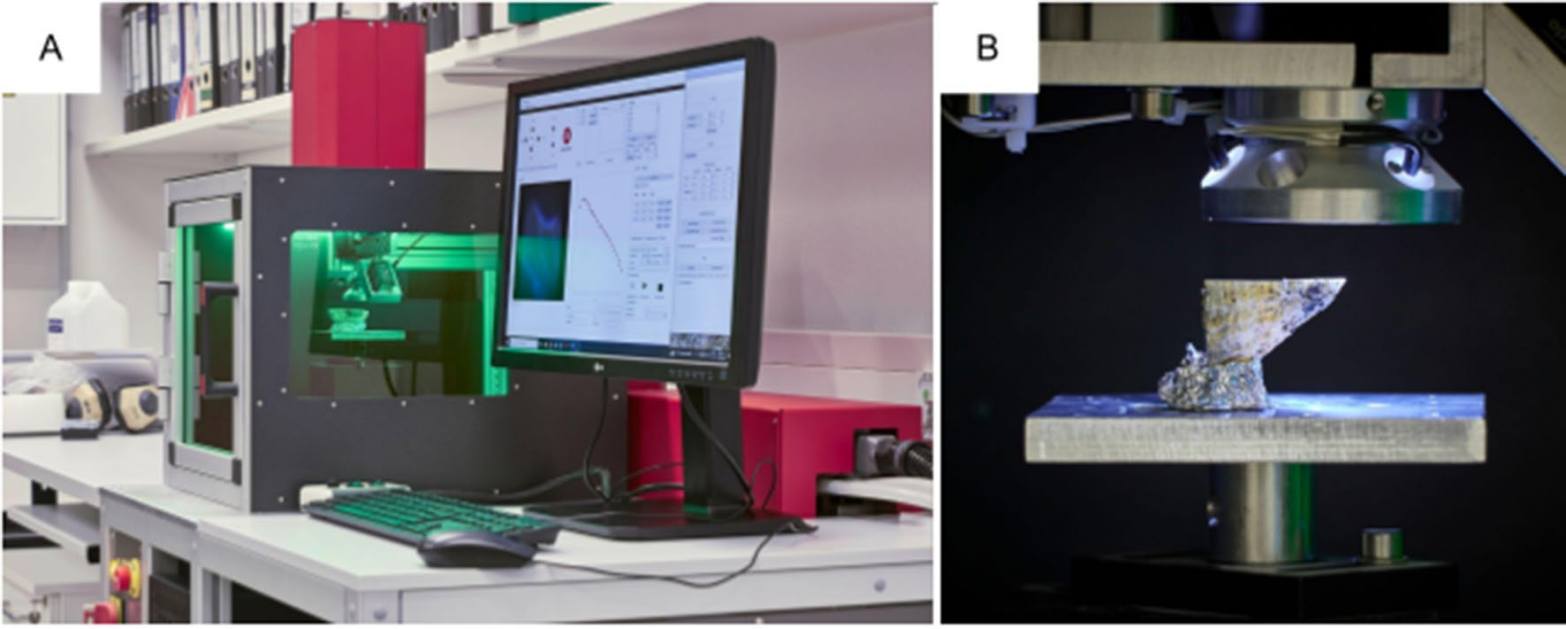
LIBS-system. (**A**) The LIBS-System workstation featuring the sample chamber (behind green glass). The laser is encased in red at the rear of the sample chamber, and its beam focused onto a location chosen via a viewing camera on the top of the sample chamber, also encased in red. (**B**) View of the open sample chamber. The laser beam enters from the top and is focused on the surface of the sample, represented by the horizontally positioned section of the mollusc shell.

The current LIBS system (Fig. 1) was developed to overcome the drawbacks of an earlier prototype, which struggled to achieve a large shell-sample throughput due to challenges faced by its slow processing speed and manual focusing^8,17^. In short, The prototype’s process of data acquisition consisted of two phases. First, a sampling path was defined with interpolated points along a set of locations. Second, using the motorised stage LIBS spectra were acquired at each pre-selected point Two measurement modes were available: the first for quickly mapping elemental ratios and the second for detailed line-scans tracking elemental changes throughout the lifetime of the mollusc. The average time spent per sampling point was about 1.7 s for mapping mode and 2.4 s for the more detailed line scan mode. Although the earlier system demonstrated LIBS' potential as an effective imaging technique, it did not achieve the desired level of efficiency.

### Components

The main concept of our system was to operate a laser, spectrometer, and XYZ-stage in concert, so that elemental images could be produced in an efficient way. We employed a pulsed DPSS (diode-pumped solid-state) and Q-switched Nd:YAG laser with a Gaussian unstable resonator (Litron; NANO-DPSS G50–100), with a repetition rate of 100 Hz and an output energy of 1.7 mJ at 1064 nm, producing 8 ns pulses. The laser beam was focused using a high-power objective (Thorlabs, LMH-10X-1064) with infrared coating (908–1130 nm) and an effective focal length of 20 mm. Visualisation of the shell surface was carried out using a CMOS camera (IDS; UI-3070CP-C-HQ Rev. 2) positioned in line with the focusing lens, sharing focus with the laser beam at a slight offset. The extent of visible sample area was ca. 3.3 by 3.3 mm with a resolution of 2056 by 1542 pixels.The shells were positioned for sampling with an XYZ motorised system, which included two translation stages (Standa Ltd.; 8MTF-102LS05) for up to 10 cm horizontal movement and one stage (Standa Ltd.; 8MVT-100-25-1) to move vertically over a distance of 2.5 cm. The exact vertical position, crucial for optimal focusing, was established using the viewing camera and a distance sensor (Micro-Epsilon; ILD-1420-50) with a resolution of 0.5 µm.

Our spectrometer consisted of a spectrograph (Andor; A-Kymera-193i-A) connected to an ICCD (intensified charge-coupled device) detector (Andor; A-DH320T-25F-03). It was equipped with a 1800 l/mm grating resulting in a 0.06 nm/pixel resolution and a spectral range of ~ 65 nm. We used the internal delay generator to set the delay time to 0.5 µs and the integration time to 1 µs.

### Data acquisition and processing

**Figure 2:**
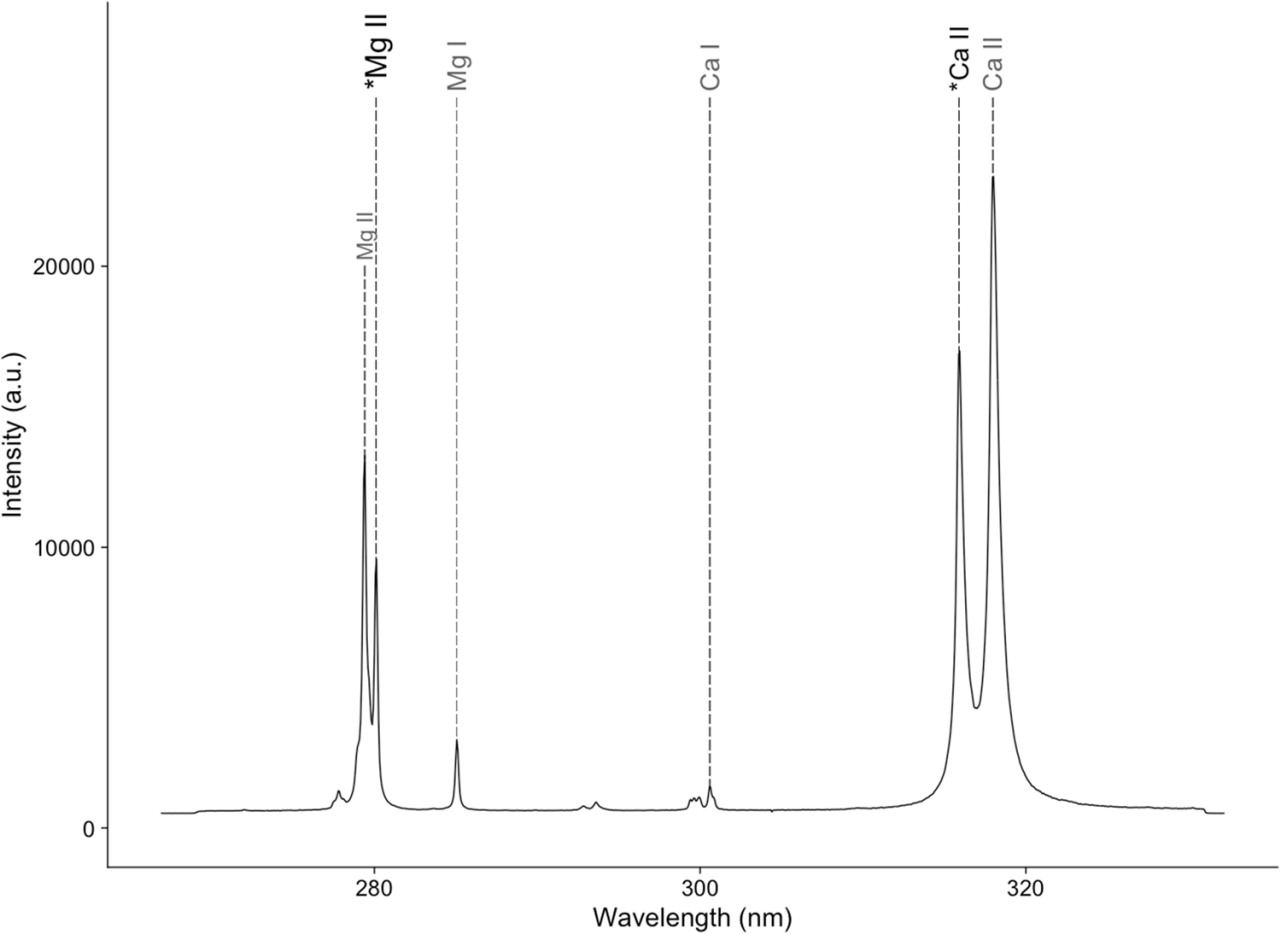
Single-shot LIBS spectrum of shell sample. Measured peaks as indicated by asterisk (*): Mg II (279.553 nm) and Ca II (315.887 nm).

**Figure 3:**
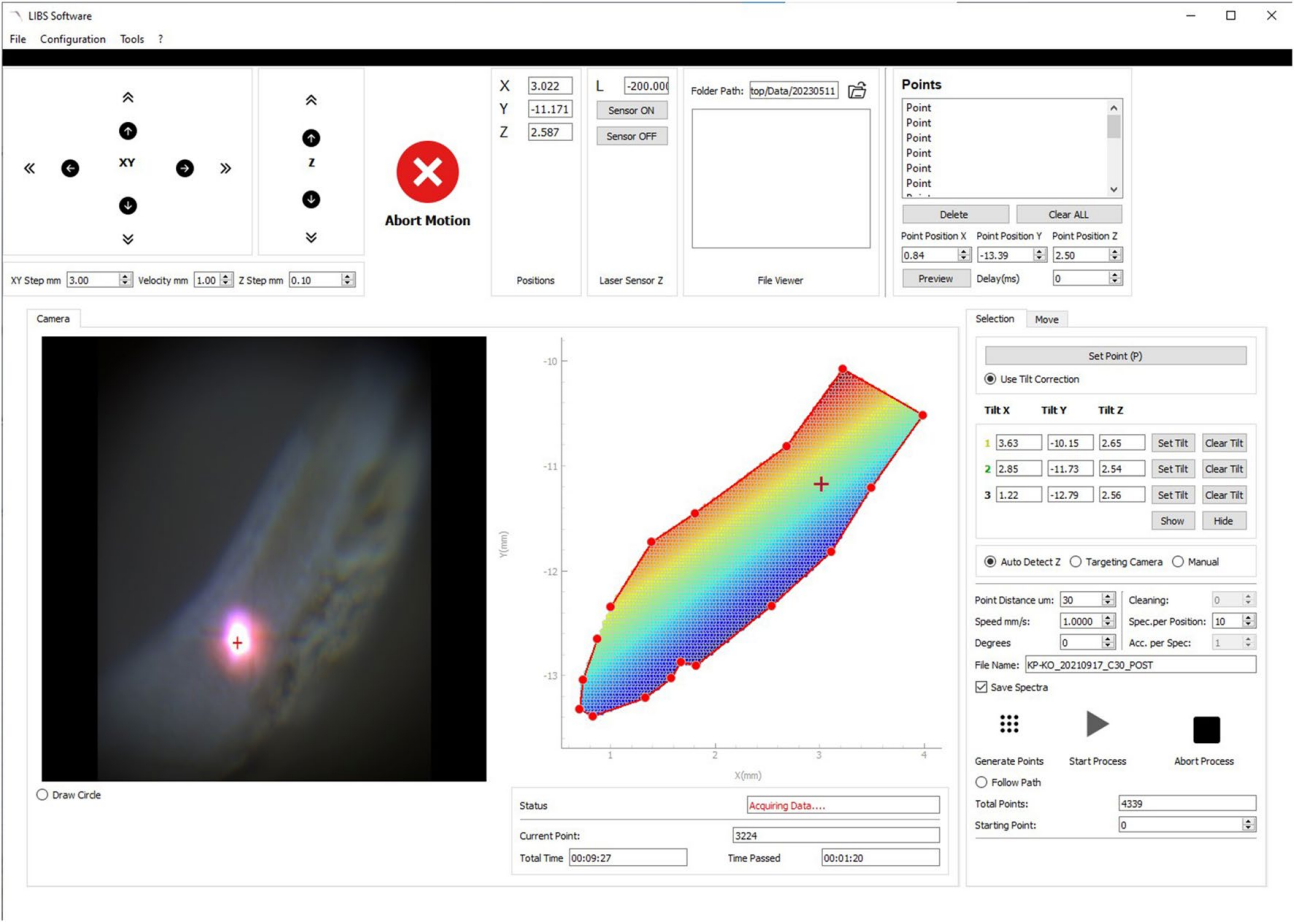
Selection of sample area. The stages are controlled via point-click on the sample viewer on the left. Sample paths and interpolated locations are shown in the centre coloured by their respective z-values. Total sample points and expected time of analysis are displayed at the bottom.

Data acquisition is performed via a custom Python software package developed by Andreas Lemonis (Biomimetic PC, Figs. 2 and 3). Two kinds of analysis modes can be selected in the software: 2-D elemental imaging or line scan analysis. Regardless of the chosen mode, the first step in any measurement involves defining the area (or path) to be analysed using a point-and-click interface. Next, to maintain consistent focus conditions while scanning the shell, the tilt of the shell surface is determined using a laser distance sensor. Using the information obtained in the previous steps, the XYZ-coordinates of each position to be measured are then interpolated at a user-defined resolution (default is 30 µm). Before measuring, the number of spectra per sample location can be set. The default values are 10 spectra per spot, with 3 of those being used for cleaning the surface. For each group of spectra, the motorised stages move the sample to the desired position. Once the stages stop, the laser and spectrometer are activated to capture the specified number of spectra before proceeding to the next position.

Each measurement’s spectrum is saved into an external file, which can subsequently be opened for spectral analysis.

**Figure 4:**
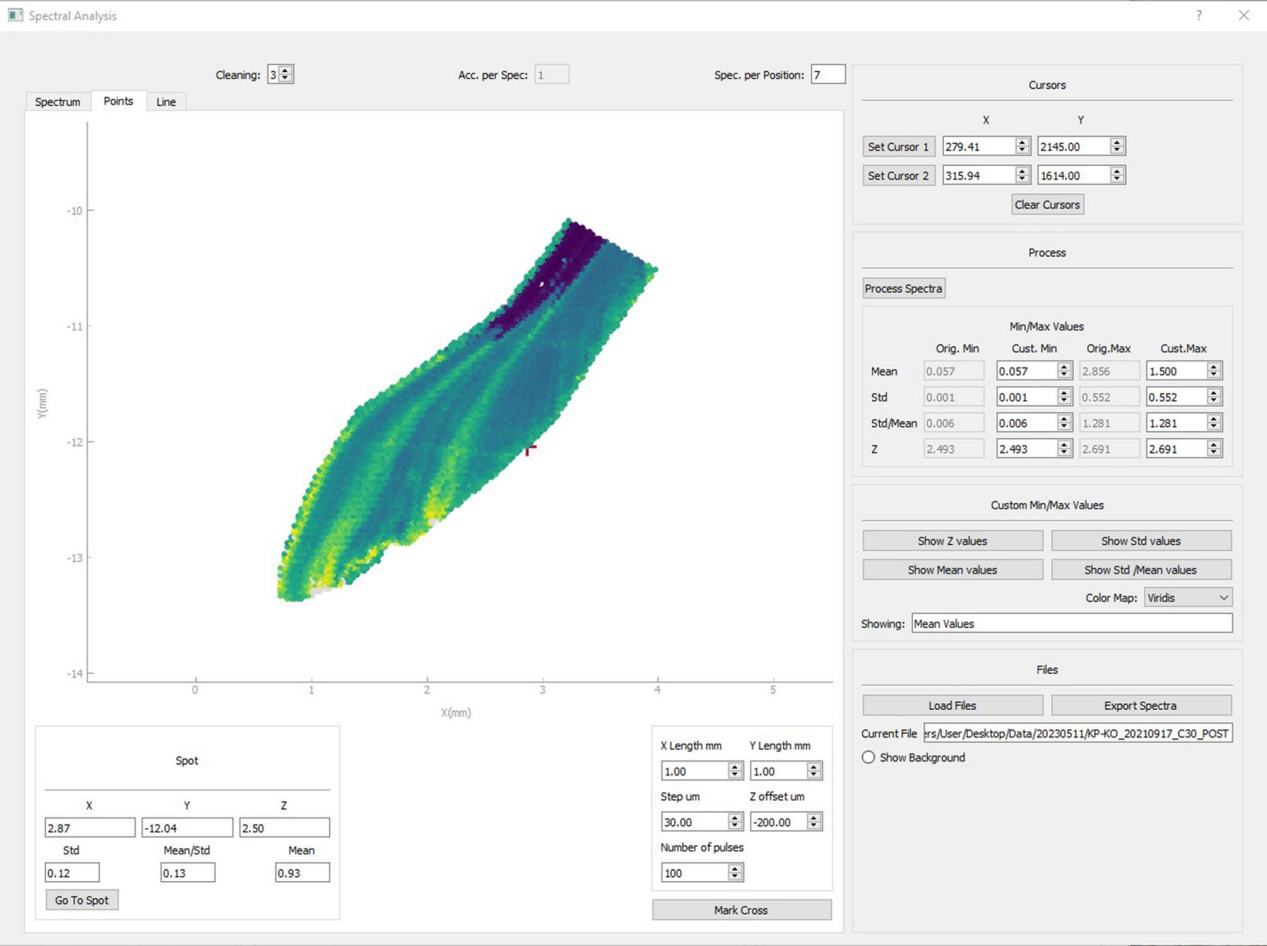

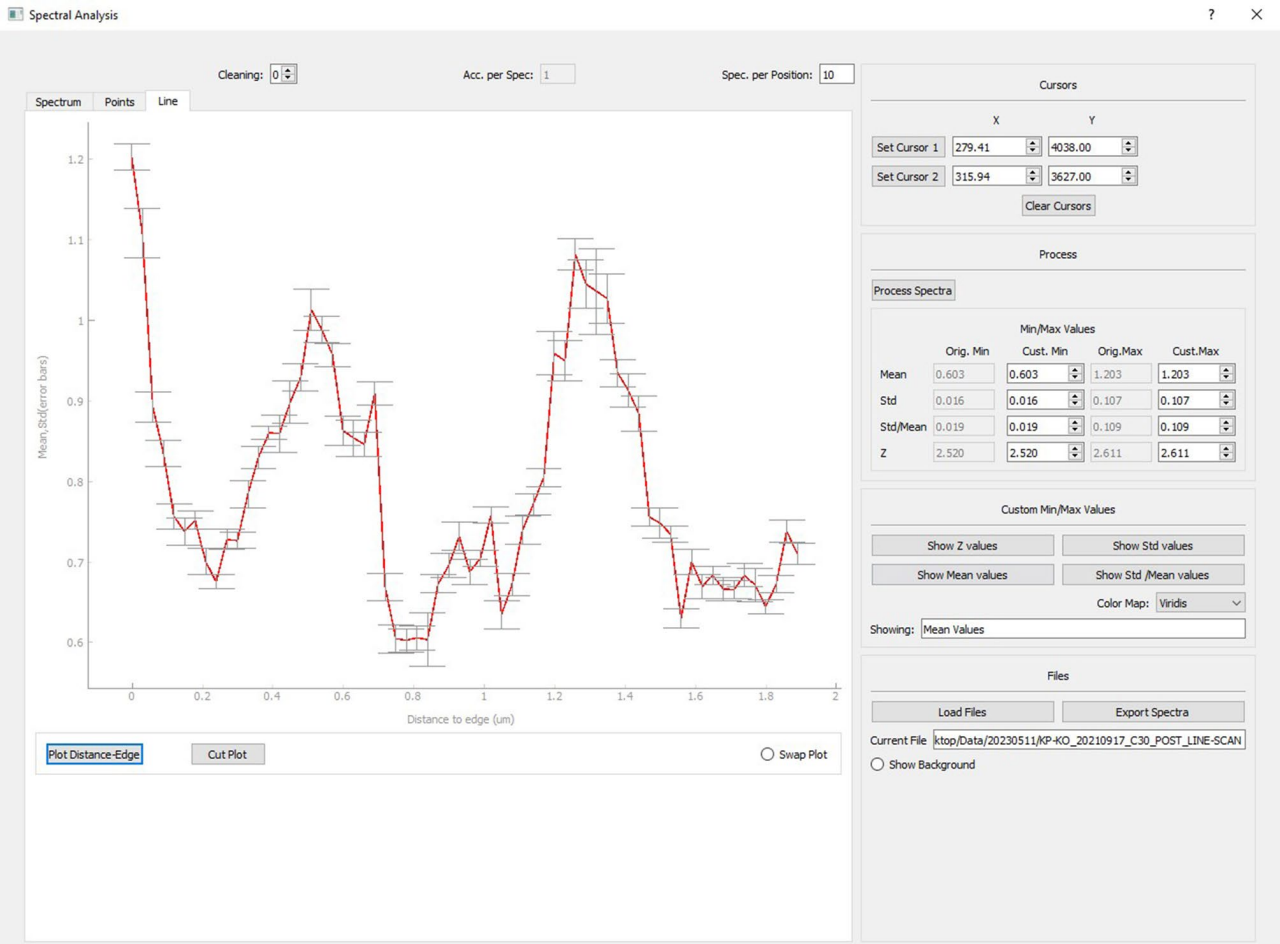
Display of elemental data. Top: an elemental image, the display-colours can be selected from a range of options. Bottom: line graph of line-scan measurement to better illustrate changes through time and provide comparative data for other geochemical proxies.

In this second step, the software calculates the ratio between the intensity values of two predetermined emissions lines and calculates the average value, standard deviation, and relative standard deviation for each individual sample spot and with the chance to discard more spectra as cleaning-shots. The results are saved in a separate CSV file (comma-separated values), including x-, y-, z-coordinates, mean ratio, standard deviation, and relative standard deviation for each sample spot. These values can then be displayed as coloured 2-dimensional map or as a line-graph (Fig. 4).

Additional points of interest, such as annual minimums or maximums within the shell record, can be chosen using a point-and-click interface. These points can be used for further measurements or marked as areas of interest for alternative sampling methods. These markings are made by selecting a specific location via the point & click interface, which is then used as the centre of a crosshair that is irradiated onto the sample surface in the form of two perpendicular arrays of sample spots. The crosshair is then visible by the naked eye and can easily be targeted for e.g. drilling or milling for carbonate powder samples used in δ^18^O-analysis^10^.

### Samples

**Figure 5:**
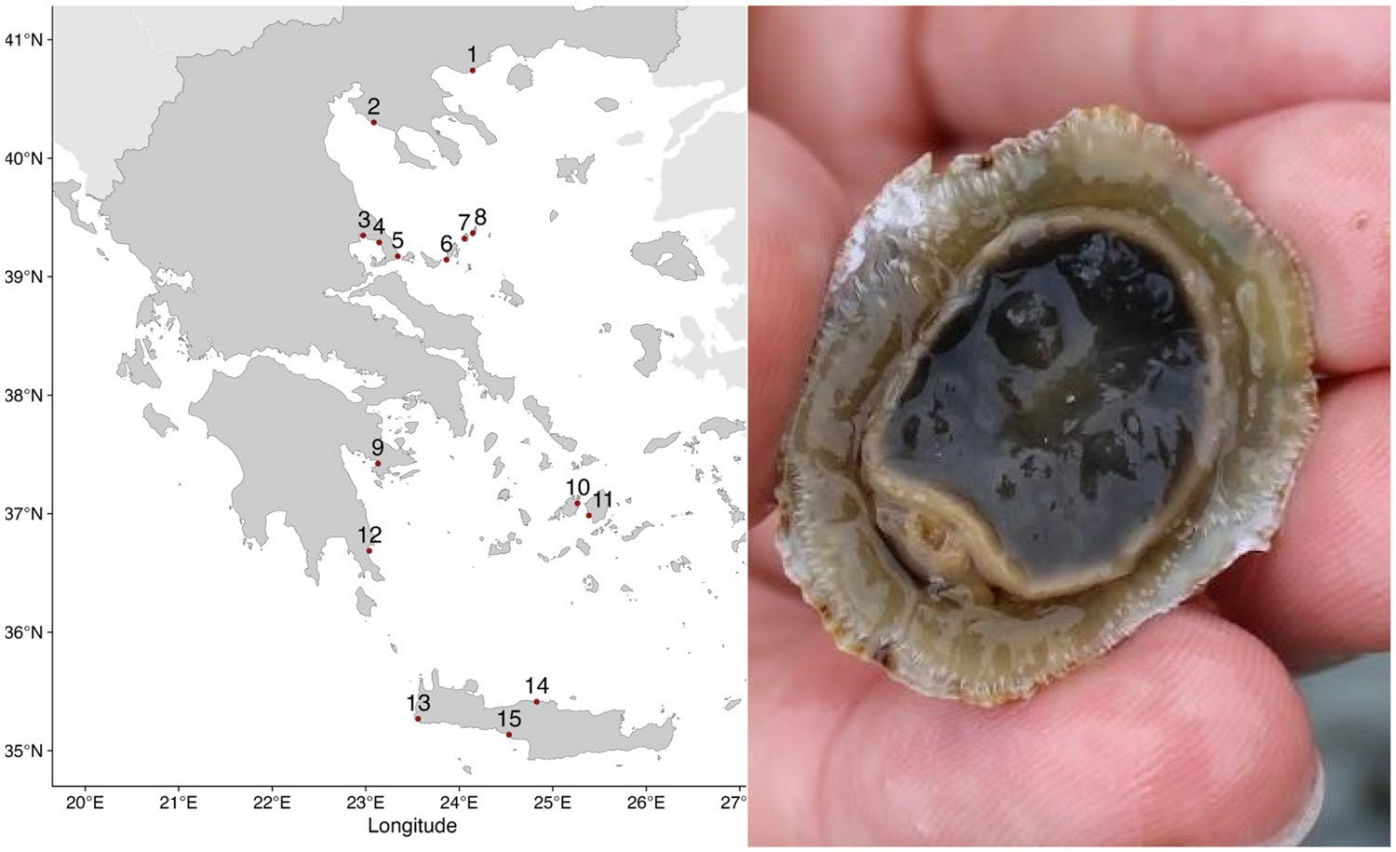
Sample collection. Locations of *Patella caerulea* across the Aegean and view of a live sample during collection.

To assess the effectiveness of the system in a real-world scenario, we have set out to image and scan a collection of marine mollusc shells (n = 101) of the species *Patella caerulea* that were collected as a reference assemblage for palaeoclimate studies of the Aegean (Fig. 5). *P. caerulea* shells possess geochemical properties that render their elemental composition as more reliable paleothermometers compared to other mollusc species^10^. Using elemental imaging as a fast and inexpensive screening tool to identify valuable climate archives and target areas of climatic interest (e.g. annual minima and maxima), of archaeological interest (i.e. season of capture), or of ecological interest (annual growth structures) thus has great potential for future research.

The modern reference collection was assembled using *Patella* shells from different regions (Table 1). So far, 101 shells have been analysed from 17 different coastal locations in Greece. Our goal was to test the shell archive under known conditions before analysing the archaeological samples. The collection started with molluscs from a nearby location with that of the archaeological shells, but it soon expanded to several other locations. The collection includes shells from Northern and Central Greece, the Peloponnese, Crete and various other islands, while all four seasons of the year are represented. A small number of limpets was collected from each location from the intertidal zone. Each sample area was at an exposed location with frequent water exchange with the open sea. Only areas 3 and 4 (Plakes, Volos and Koropi, Pagasetic Gulf) were less exposed, but still not affected by changes in water chemistry as would be expected for e.g. an estuary environment. As such, sea surface temperature ranges were similar between sites from ~ 14 to ~ 26 °C for the northern Aegean and from ~ 16 to ~ 26 °C for south of Crete.

**Table 1:** List of analysed shell specimens.

No. on map	Site	Site-code	Specimens	100 µm	30 µm
1	Pyrgos Apollonias, Kavala	PAPK	2	2	2
2	Νea Kallikrateia, Chalkidiki	NKC-M	4	4	7
3	Plakes, Volos	V-P	3	3	2
4	Koropi, Pagasetic Gulf	KO-PG	5	5	5
5	Katiyorgis, Volos	V-KB	6	6	7
6	Patitiri, Alonnisos	PA	5	5	6
7	Agios Petros, Kyra Panagia	KP-AP	15	15	17
7	Kokkino bay, Kyra Panagia	KP-KO	4	4	6
8	Southern dock, Youra	Y-S	8	8	11
8	Western dock, Youra	Y-W	12	11	19
9	Paralia, Franchthi	FR-P	23	22	36
10	Ysterni, Paros	GYP	2	2	3
11	Alyko, Naxos	AN	3	3	5
12	Monemvasia, Laconia	ML	5	5	5
13	Kedrodasos, Chania	K-CH	1	1	0
14	Glaros, Rethymno	RE-G	1	1	1
15	Ligres, Rethymno	RE-L	2	2	2
Total		17	101	100	134

Shell specimens were sampled on various resolutions (100 µm and 30 µm) depending on the size of the areas of interest.

The shells were cleaned solely with water and dried before they were sectioned lengthwise using a diamond-coated blade on a slow-speed saw (Buehler; IsoMet 1000). All sections followed the main growth axis across the apex, revealing a triangular shape with two growth edges on the sides (anterior and posterior) and the apex in the centre. Shells were left unpolished, apart from specimens collected near Franchthi (FR-P_20210515) A to D, which also underwent optical microscopic and/or SEM analysis. Grinding and polishing was carried out using metallographic grit paper (P800, P1200, P2400) and a 3 µm diamond suspension (Buehler; MetaDi Combo). Prior to their analysis using LIBS, shells were cleaned with ethanol and a microfiber cloth. For the SEM analysis, specimen FR-20210515-C was sputter-coated with a 3 nm-thick gold film and examined using a JSM-6390 LV SEM (JEOL Ltd.) at the University of Crete.

## Results and discussion

### LIBS

In total, 101 shell specimens were successfully analysed (Table 1). The sampling strategy was to collect an overall image of each shell section using a 100 µm resolution and then to focus on one or both growth edges using 30 µm resolution if necessary. In nearly all cases, this step proved necessary, and the higher resolution images consistently offered greater assistance in interpreting the shell record. This is likely due to the species size and growth rate. Other species might well work better using different resolutions or sampling parameters.

The time difference between these two resolutions is not negligible, despite the other parameters (i.e. laser frequency, spectra per point) are identical. The larger gaps between sampling spots also increased the acquisition time, resulting in 4.9 points analysed per second (296 points per minute) at 100 µm resolution and 7.6 points analysed per second (459 points per minute) at 30 µm resolution. This is a 10 to 15 times faster acquisition to the previous system^8^. This speed could be increased to 12 sample spots per second by lowering the number of spectra per spot to 1 (as carried out by^16^) and forgoing the cleaning step. The number of spectra per second, however, is then reduced from 76 to 12, lowering the amount of data acquired during this period and lowering the overall robustness of the dataset. Consequently, the acquisition speed represents a balance between data quality and the necessary efficiency for the given task. Ultimately it is also restricted by the distance between sample spots, the movement speed of the motorised stage, the rate of repetition of the laser, and the readout time of the ICCD camera. The 2-dimensional view of the elemental distribution allowed us to verify whether there is one shared Mg/Ca ratio across one growth increment or whether there is anomalous intra-increment variation, as has been shown elsewhere8. If there is variation, traditional line scans might lead to the interpretation of these anomalies as climatic information, further emphasising the need for 2D-scanning. We found that around half of the specimens had these variations with no obvious reason (e.g. age, location, season, size) for why they occur (see supplementary information).

**Figure 6:**
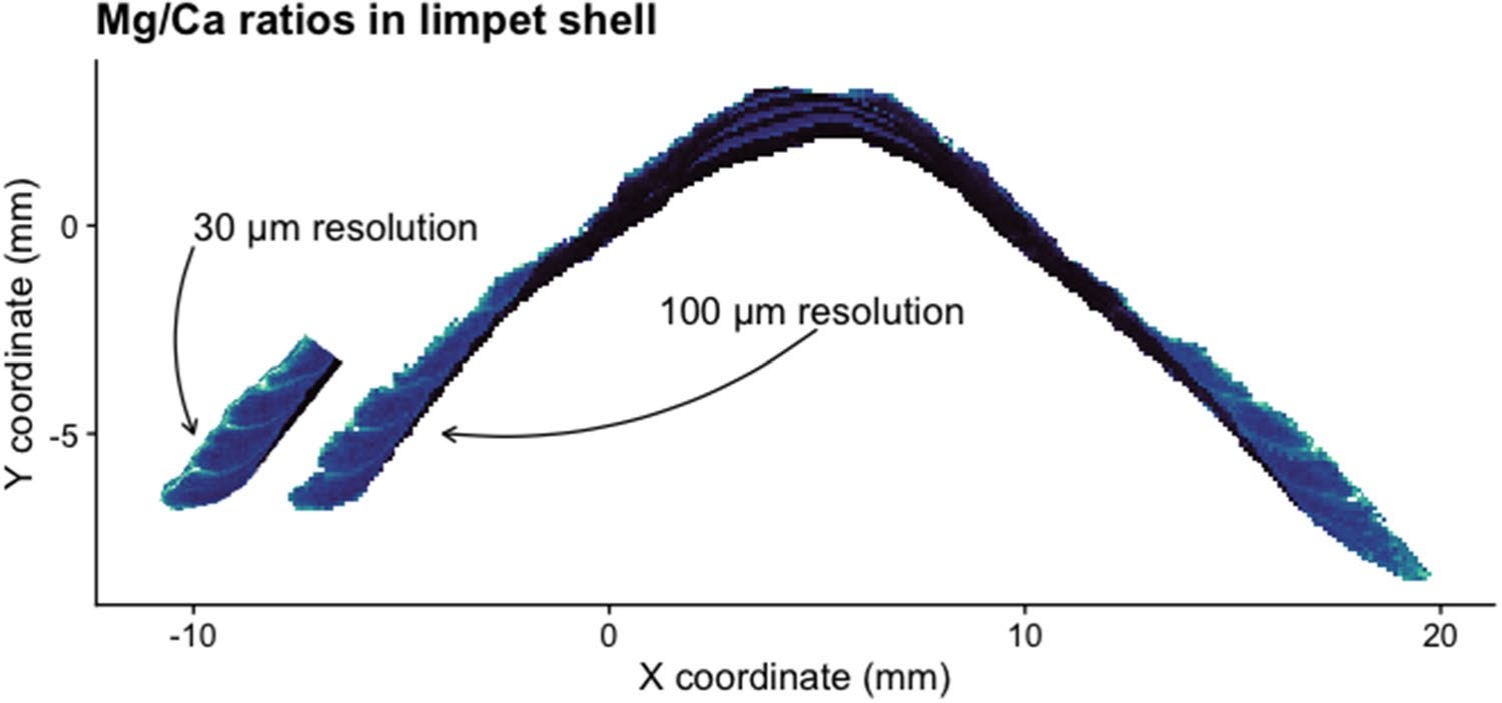
Elemental image. Typical result from a shell specimen (KP-AP_F), where the 100 µm resolution image provides an overview of the shell section and the 30 µm resolution image allows for a more detailed view of the area of interest.

**Figure 7:**
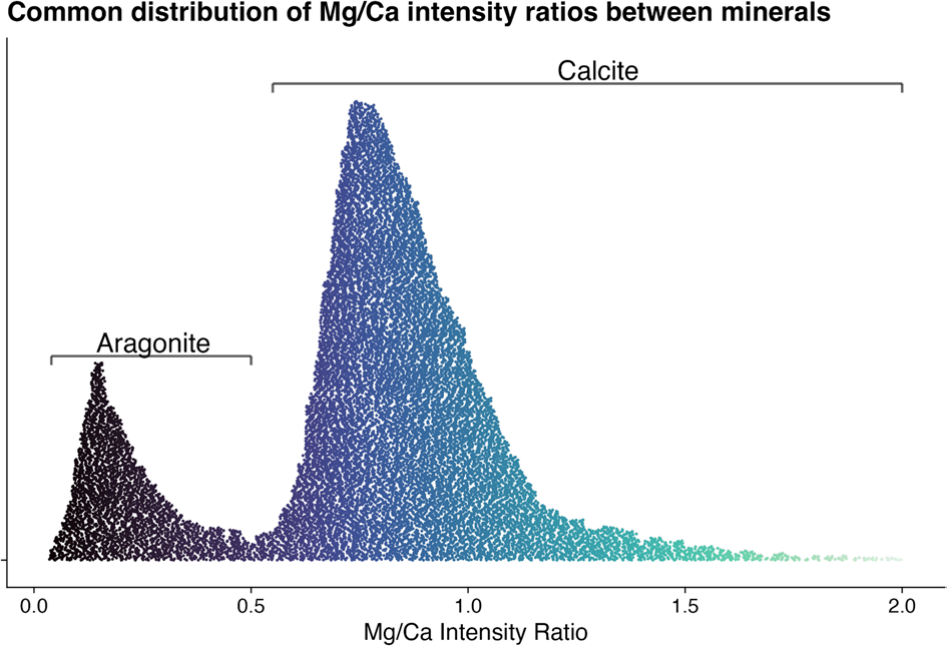
Distribution of Mg/Ca ratios. The lower Mg/Ca ratios are commonly found in the aragonitic parts of the shells, while calcitic parts produce higher Mg/Ca ratios.

**Figure 8:**
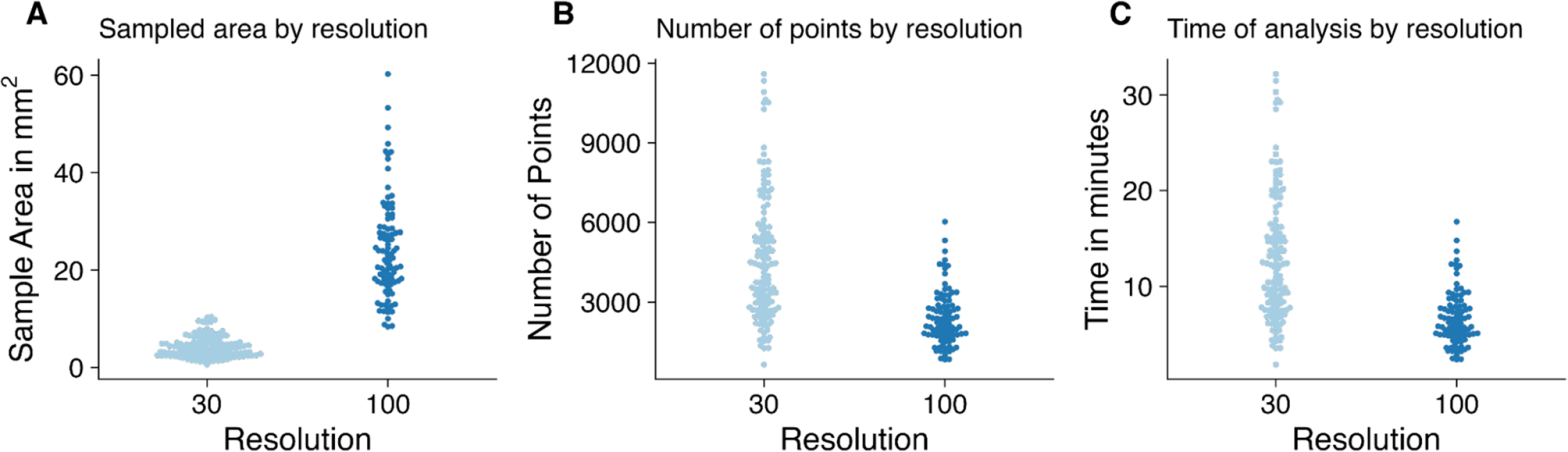
Comparison of imaging efficiency. (**A**) Area covered by the two kinds of resolution. (**B**) Sample points per image. (**C**) Time of analysis.

Shells of the genus Patella have a calcitic part on the rim and an aragonitic part in the centre^18^. The produced images follow a similar pattern, with an aragonitic centre in each shell section and two calcitic rims on the sides (Fig. 6). The calcitic parts of the shell section usually fall into the higher ranges of 0.5–2.0, while the aragonitic parts of the shell section almost exclusively produce intensity ratios below 0.5 (Fig. 7). The calcitic rims point towards the posterior (less steep angle) and the anterior (steeper angle) ends of the shell and contain higher resolution climatic records, which is why these have been targeted for subsequent imaging using a 30 µm resolution. Thus almost every shell section was sampled multiple times. These higher resolution images frequently encompassed smaller areas (30 µm average: 4.2 mm^2^, 100 µm average: 24.0 mm^2^) but contained a larger amount of points (30 µm average: 4648 points, 100 µm average: 2402 points), consequently demanding more time (30 µm average: 12.9 min, 100 µm average: 6.8 min) (Fig. 8).

### SEM—evaluation of the LIBS analysis on the shell surface

**Figure 9:**
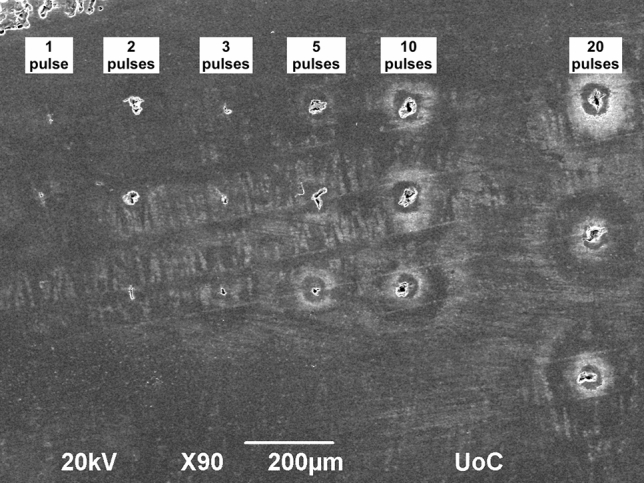
Image of LIBS craters. Different numbers of laser pulses leave various degrees of impact on a polished shell surface.

The SEM experiments demonstrated the varying degrees of impact that LIBS analysis exerts on the shell surface (Fig. 9). While a single pulse crater lies well below 10 µm in diameter, two pulses cause craters with diameters between 10 and 30 µm, 5 pulses between 10 and 50 µm, ten pulses between 30 and 50 µm, and 20 pulses also between 30 and 50 µm. The overall shapes and sizes of the craters are variable with large fissures, which explain the large and overlapping ranges of destruction between the different amounts of pulses. A crater of two pulses can be as destructive as 10. Equally, five pulses can cause a crater as small as two pulses can. These fissures are most likely caused by the underlying differences in the shell's crystal fabric structure, which is more or less prone to shattering and fragmenting in various parts. Despite this underlying factor of the crystal fabric, there is consistently a deeper hole inside the crater, measuring 10–15 µm, which we expect to be the area predominantly irradiated and converted into plasma. The explosive dynamic of that reaction then causes the secondary destruction of the surrounding crystal fabric, which increases with lower depths (i.e. repeated irradiation by multiple pulses).

### Sampling methods

**Figure 10:**
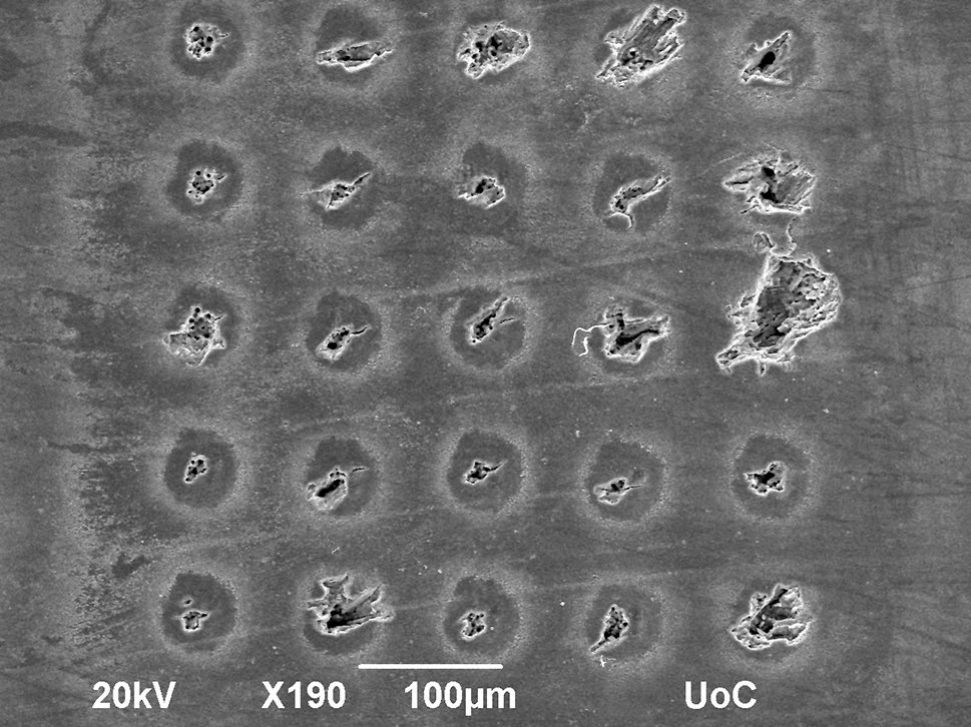
SEM image of sampling at 100 µm resolution. The impact of the LIBS analysis on the shell surface is visible with some craters being many times the sizes of others. The underlying structure of the crystal fabric is likely the main cause for the variability of fragmentation.

We additionally assessed the effect on the shell surface of the two applied sampling resolutions (100 µm and 30 µm, each with 10 pulses per location). With the 100 µm resolution the shapes and dimensions of the craters vary and occasionally exceed 50 µm and even reach 100 µm, almost overlapping with other sample locations (Fig. 10). The redeposited materials form a halo around the craters and are ~ 50 µm in diameter.

**Figure 11:**
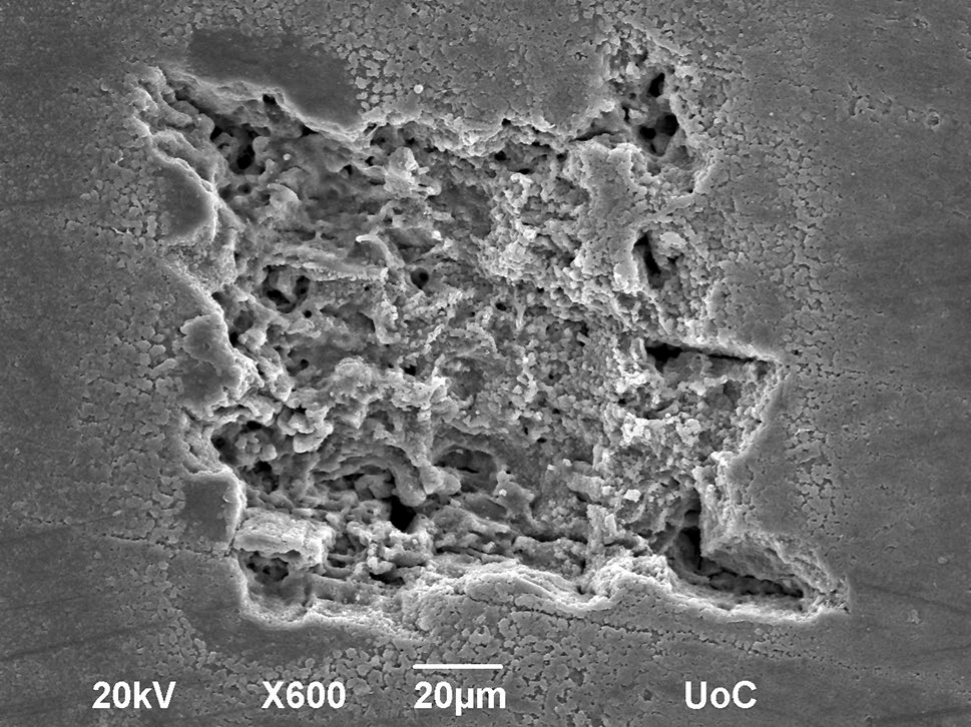
SEM image of sampling at 30 µm resolution. With some craters being more than 30 µm large, their individual borders overlap and become one continuous rough surface.

Using a 30 µm sampling resolution, the borders between sample locations vanish, as craters, fissures, and redeposited material begin to overlap (Fig. 11). However, individual measurements shown in Fig. 9 indicate that the sampled material itself does not overlap between sample locations at this resolution. Redeposited material would cover other sample locations, but should be removed by the cleaning pulses and their influence on the overall value should thus be negligible.

### Edge variability

**Figure 12:**
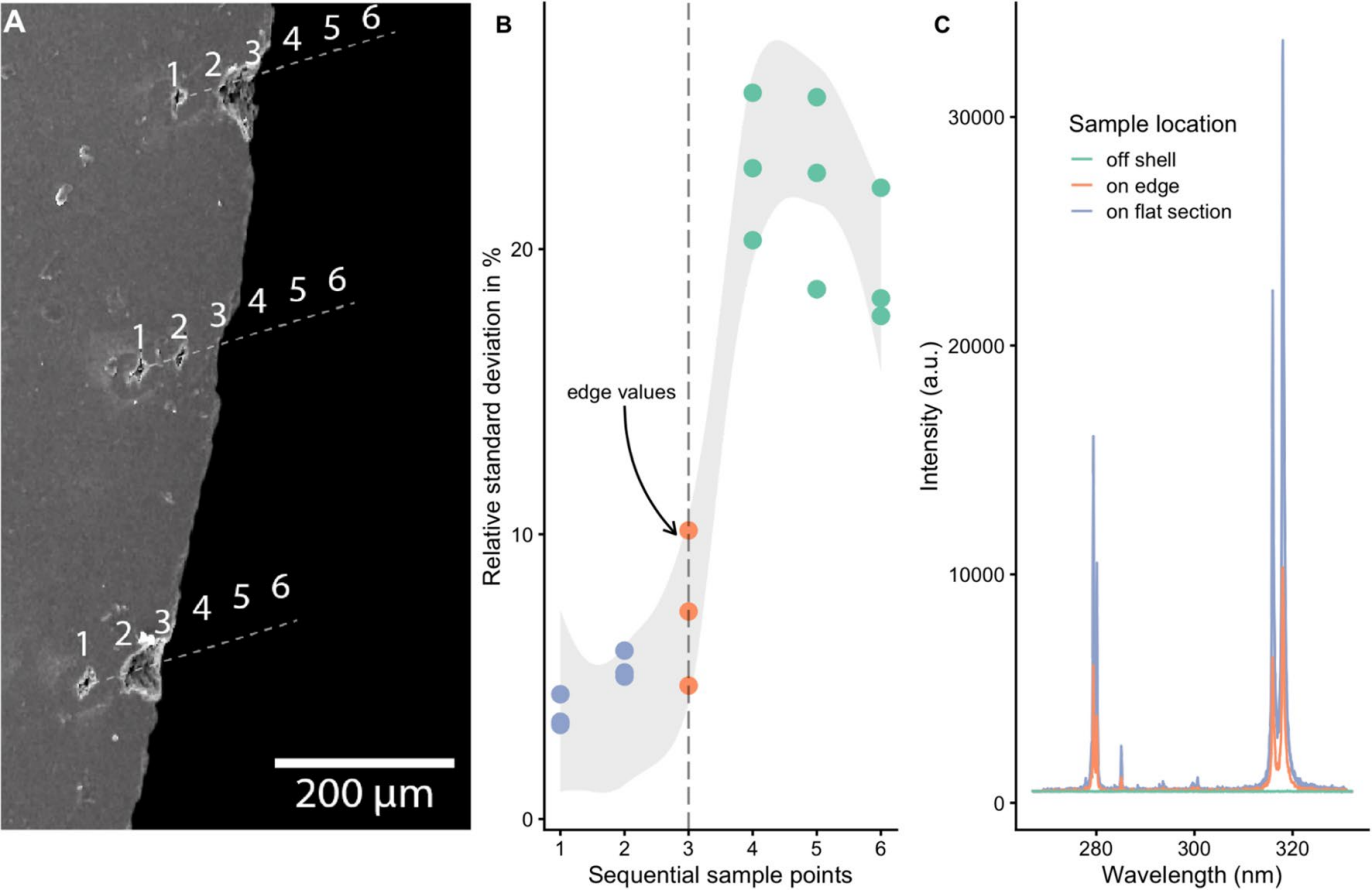
(**A**) Edge values and SEM image of their location. (**B**) A graph of the relative standard deviation in relation to the sample location demonstrates that edge values towards the shell edge (i.e. sample points 1, 2, and 3) increasingly exhibit less accurate values for intensity ratios, and when the focus moves off the edge (i.e. sample points 4, 5, and 6) the relative standard deviation more than doubles. (**C**) A graph displaying typical spectra at the various locations and explaining the reason for the more variable edge values (in orange). The lower intensity of their spectra compared to sample locations on the shell (blue) weaken the overall signal and thus increase the uncertainty of the Mg/Ca ratio values. The high degree of relative standard deviation off the shell can be easily explained by the low light emission, as there is no substrate in the laser beam's focus.

We further investigated the effect of LIBS measurements near the shell edge and its influence on the quality of produced data. Mg/Ca (a proxy for palaeotemperature) data in these edge locations is of particular interest to archaeologists, who want to reconstruct the specimen’s time of death, as it can indicate the time of site habitation or reveal patterns of past subsistence economy. A quality evaluation of this location is, therefore, beneficial for subsequent interpretations. Comparing the craters towards the shell edge, the first, most distant, location is comparatively small, producing values with a relative standard deviation of ~ 5%. These values increase the closer the measurements approach the shell edge. In the second measurement locations, the relative standard deviation increases slightly and in two cases the craters expand all the way towards the shell edge, potentially causing less accurate information as the focusing of the laser beam is inhibited. That said, it is possible that this expansion of the crater only happened after the analysis of the third measurement on the very edge. These third measurements (Fig. 12 orange) have a higher relative standard deviation reaching over 10%. At these locations on the very edge, the laser beam is no longer entirely focused on the shell section's surface but also encompasses the surrounding air and potentially irradiates parts of the shell lying several millimetres below the edge. This impact is increased as the sample locations move away from the shell surface and off the edge, reaching values for relative standard deviation above between 15 and 25%. These high values derive from measuring solely the background. As such, a more accurate way of filtering out problematic sampling locations would be to exclude spectra below a certain intensity threshold.

## Summary

In this overview, we have provided a practical example of a new LIBS-system for elemental imaging of carbonate shells using a modern assemblage used in current archaeological and palaeoclimatological research. In comparison to previous systems with similar aims^8^, the acquisition process has been accelerated by a factor of 10–15 to ~ 76 spectra per second at 30 µm. We have also improved the software's user-friendliness by incorporating a point-and-click interface and confining the laser beam within the housing, thus providing a safer working environment.

SEM analysis of the LIBS-craters showed the micrometer scale destructiveness of the method and the size of the irradiated area, which is being analysed, to be 10 to 15 µm, the equivalent of several days to a week in the climate record. Furthermore, we were able to better understand the errors that are being introduced when sampling along the shell edge, where matrix effects occur more strongly than on the flat surface of the shell section.

Future analytical steps should include the impact of laser irradiation through LIBS on other palaeoclimate proxies such as stable carbon and oxygen isotope analyses, and whether it introduces significant and/or predictable biases. Our current suggestion is to grind and polish prior to sampling for isotope analysis or to discard initial surface parts of the drilled powder samples.

The speed, ease, and repeatability of analyses is a major benefit of LIBS, allowing researchers to reassess sampling parameters, such as resolution or spectra taken per location, further enhancing the analytical detail of palaeoclimatic data. An option that is not available to many other, more destructive or time-intensive methods.

## Data Availability

All data needed to evaluate the conclusions in the paper are present in the manuscript. All underlying data files, can be found in an OSF repository at https://osf.io/jur2d/ as well as a GitHub repository at https://github.com/Niklas-palaeo/LIBS_System-2023/. The data itself is citeable using this https://doi.org/10.17605/OSF.IO/JUR2D. We are committed to promoting transparency and reproducibility in our research. Any data not already included in the manuscript can be provided by the corresponding author upon reasonable request. We believe in the importance of open science and are willing to share our data to facilitate further investigations and collaborations. Please contact [corresponding author's email] for inquiries regarding data availability.
